# A Barrier to Defend - Models of Pulmonary Barrier to Study Acute Inflammatory Diseases

**DOI:** 10.3389/fimmu.2022.895100

**Published:** 2022-07-07

**Authors:** Anna Herminghaus, Andrey V. Kozlov, Andrea Szabó, Zoltán Hantos, Severin Gylstorff, Anne Kuebart, Mahyar Aghapour, Bianka Wissuwa, Thorsten Walles, Heike Walles, Sina M. Coldewey, Borna Relja

**Affiliations:** ^1^Department of Anaesthesiology, University of Duesseldorf, Duesseldorf, Germany; ^2^L Boltzmann Institute for Traumatology in Cooperation with AUVA and Austrian Cluster for Tissue Regeneration, Vienna, Austria; ^3^Department of Human Pathology , IM Sechenov Moscow State Medical University, Moscow, Russia; ^4^Institute of Surgical Research, University of Szeged, Szeged, Hungary; ^5^Department of Anaesthesiology and Intensive Therapy, Semmelweis University, Budapest, Hungary; ^6^Experimental Radiology, Department of Radiology and Nuclear Medicine, Otto-von-Guericke University, Magdeburg, Germany; ^7^Research Campus STIMULATE, Otto-von-Guericke University, Magdeburg, Germany; ^8^Department of Anaesthesiology and Intensive Care Medicine, Septomics Research Centre, Centre for Sepsis Control and Care, Jena University Hospital, Jena, Germany; ^9^Department of Thoracic Surgery, Magdeburg University Medicine, Magdeburg, Germany; ^10^Core Facility Tissue Engineering, Otto-von-Guericke-University, Magdeburg, Germany

**Keywords:** air-liquid, 2D, 3D, co-culture, ALI, organoid, LOAC, PCLS

## Abstract

Pulmonary diseases represent four out of ten most common causes for worldwide mortality. Thus, pulmonary infections with subsequent inflammatory responses represent a major public health concern. The pulmonary barrier is a vulnerable entry site for several stress factors, including pathogens such as viruses, and bacteria, but also environmental factors e.g. toxins, air pollutants, as well as allergens. These pathogens or pathogen-associated molecular pattern and inflammatory agents e.g. damage-associated molecular pattern cause significant disturbances in the pulmonary barrier. The physiological and biological functions, as well as the architecture and homeostatic maintenance of the pulmonary barrier are highly complex. The airway epithelium, denoting the first pulmonary barrier, encompasses cells releasing a plethora of chemokines and cytokines, and is further covered with a mucus layer containing antimicrobial peptides, which are responsible for the pathogen clearance. Submucosal antigen-presenting cells and neutrophilic granulocytes are also involved in the defense mechanisms and counterregulation of pulmonary infections, and thus may directly affect the pulmonary barrier function. The detailed understanding of the pulmonary barrier including its architecture and functions is crucial for the diagnosis, prognosis, and therapeutic treatment strategies of pulmonary diseases. Thus, considering multiple side effects and limited efficacy of current therapeutic treatment strategies in patients with inflammatory diseases make experimental *in vitro* and *in vivo* models necessary to improving clinical therapy options. This review describes existing models for studyying the pulmonary barrier function under acute inflammatory conditions, which are meant to improve the translational approaches for outcome predictions, patient monitoring, and treatment decision-making.

## Introduction

Main functions of the lung as oxygen delivery and decarboxylation require unique structures within the lung tissue, providing physiological oxygen levels necessary for all human cell functions. Pulmonary functions are based on gas-exchanging units, i.e. alveoli mainly covered by epithelial cells. Together with the pulmonary capillary endothelium and the basal lamina inbetween, these alveolar epithelial cells (AECs, comprising two main cell types: pneumocytes type I, and pneumocytes type II) form a tight barrier. This so-called air-blood barrier enables an efficient gas exchange between air and blood, prevents the influx of protein-rich fluid into alveolar spaces, and protects the body from pathogenic microorganisms and environmental pollutants (1). Undisturbed gas exchange is crucial for maintaining oxygen supply and cell survival. Therefore air-blood barrier dysfunction is a critical pathological event leading not only to lung injury but potentially to patient morbidity and death (2, 3). Physiologically, maintenance of the barrier is guaranteed by anti-inflammatory reactions, involving complex interactions between and among immune cells and structural lung cells (4, 5). In particular, the interaction of AECs with alveolar macrophages (AMs) maintains a balance in the air-blood barrier, and enables self-repair processes at and of the barrier (6). However, aerogene stimuli like bacterial, viral, and fungal agents, as well as inhaled toxic particles and physical stress factors as pressure waves, trigger local and systemic pro-inflammatory reactions mediated by immune cells, such as dendritic cells (DCs) and AMs (7–9). This provoked release of pro-inflammatory mediators (e.g. tumor necrosis factor (TNF)-α, interleukin (IL)-1β, IL-8) as well as the production of reactive oxygen and nitrogen species (RONS) (10, 11) outweigh the anti-inflammatory processes at the air-blood barrier. Pulmonary inflammation plays an important role in the pathogenesis of acute pulmonary diseases, like pneumonia, as well as in the development and progression of chronic inflammatory disorders such as asthma and chronic obstructive pulmonary disease (COPD) (12, 13). Moreover, damage of AECs contributes to the development of acute respiratory distress syndrome (ARDS), one of the most destructive inflammatory processes occurring in the lung (14). Due to the vital role of the lungs in gas exchange, excessive inflammation in the lung tissue often leads to life-threatening conditions (15).

Lung injury is the primary cause of patient morbidity and mortality in many diseases, including coronavirus disease (SARS-CoV-2). The histopathological studies demonstrated a significant and profound alveolar damage and pneumonia, which progresses to ARDS and in the long-term to lung fibrosis in a multitude of patients (16). Meanwhile, the clinical manifestation of SARS-CoV-2 is paralleled by a sudden surge in pro-inflammatory cytokines known as “cytokine storm,” parenchymal loss, immune infiltration, and fluid-filled alveoli, altogether potentially resulting in an acute pulmonary failure and death (17, 18). Thus, to develop safe and effective therapies for infectious and inflammatory pulmonary diseases, it is crucial to understand cell-type-specific changes as well as the mechanisms and sequalae of the humoral immune responses in the lung. Additionally, reliable, well-defined experimental models with standardized conditions can provide a high-throughput therapeutical screening tools. Apart from *in vivo* models, several *in vitro* approaches have been developed to mimic the response to both pro-inflammatory stimulation and anti-inflammatory treatment, which range from simple cellular monolayers, multicellular models with primary or immortalized cell lines, primary tissue-derived organoids, alveolosphere cultures, to 3D multicellular systems of lung epithelial tissue. These approaches serve as a versatile and realistic toolkit for modeling and studying the pulmonary barrier function, and as screening tools for drug efficiency and safety. In the current review, we highlight different *in vivo* and *in vitro* models to examine pulmonary barrier, particularly during acute inflammatory conditions leading to lung injury.

## The role of oxidative stress in lung homeostasis and barrier injuries

Depending on the type of RONS that is generated, they can either cause oxidative damage to biomolecules and biological structures or contribute to intracellular signaling cascades (19). Early phase RONS, such as superoxide, hydrogen peroxide and nitric oxide (NO) are predominantly involved in signaling, while secondary RONS, such as peroxynitrite (ONOO) and hydroxyl radical are associated with oxidative damage to biological structures (19). Excessive production of RONS and oxidative damage may initiate a loop forward vicious cycle causing further damage to host cells (20, 21). This is due to the fact that induction of oxidative stress can further accelerate inflammation *via* NF-kB mediated pathway (22). Enhanced RONS generation and subsequent activation of oxidative stress-related pathways are the key processes causing damage to the pulmonary barrier *via* several specific pathological mechanisms (23). Thimmulappa et al. suggested eight oxidative stress-related mechanisms underlying pulmonary barrier damage: (1) lipid peroxidation, a process which disturbs the integrity of lipid bilayer of epithelial cells increasing their permeability, (2) RONS-induced activation of inflammatory response, (3) RONS-mediated activation of pathways involved in programmed cell death and more recently ferroptosis (24), (4) oxidative stress-mediated mitochondrial dysfunction, (5) endoplasmic reticulum stress-mediated disruption of protein synthesis (6) RONS-mediated epigenetic modifications *via* direct interaction with DNA/RNA, (7) activation of profibrotic mechanisms associated with endothelial cell dysfunction, and (8) airway mucus hypersecretion (23). AMs play a key role in the induction of these RONS-mediated pathways, since they are the first immune cells dealing with pathogens or foreign substances in the lung. They respond to bacterial/viral pathogens or their components such as pathogen-associated molecular pattern (PAMPs) or to substances released from damaged host cells (damage-associated molecular pattern, DAMPs) by releasing pro-inflammatory cytokines such as CXCL8, which upon release recruit neutrophils, the strong generator of ROS, into the lungs. AMs, AECs and neutrophils generate RONS predominantly *via* activation of NADPH oxidase (NOX)-2 located in the cellular membrane of these cells (25, 26). The activity of this enzyme is tightly linked to the intracellular generation of mitochondrial RONS. In addition to RONS derived from NOX and mitochondria, also xanthine oxidase and uncoupled NO-synthase (NOS) can contribute to excessive RONS generation occurring upon inflammatory conditions (27). Activation of both NOX-2 and NOS in turn stimulates the production of mitochondrial RONS and their release in cytoplasm *via* two discrete feed-forward loops (28). This interplay between intracellular and extracellular RONS generators can be beneficial in elimination of bacteria (28). However, the overproduction of intracellular RONS uncouples endothelial isoform of (e)NOS, which leads to formation of harmful peroxynitrite (ONOO^-^) instead of NO. ONOO^-^ further aggravates oxidative stress and endothelial dysfunction (29). In addition to PAMPs and DAMPs, angiotensin (Ang)-II also activates NOX-2 *via* protein kinase (PK)C pathway (30), which leads to excessive RONS generation and pulmonary barrier damage particularly during SARS-CoV-2 infection. Therefore, oxidative stress and exaggerated inflammation are the major contributors to pulmonary barrier disruption which plays critical role in the pathogenesis of pulmonary diseases, including ARDS. Although pulmonary immune and oxidative homeostasis will not be further addressed in this review, it is of utmost importance to note that the pulmonary homeostasis is maintained by a complex network of tissue-resident cells as well as recruited immune cells. Incorporation of this complexity and orchestration into *in vitro* models is highly challenging but essential to determine und identify novel strategies for disease prevention and treatment. Therefore, it is essential to unfold this complexity by understanding different cellular and factoral entities of the pulmonary barrier and their incorporation into the experimental models.

## Special features of the microcirculatory pulmonary barriers

Pulmonary microcirculation is an important component of the pulmonary barrier, and it is characterized by unique features differing from those of the systemic microvasculature. The pulmonary circulatory bed belongs to the low-resistance circulation area that must adopt to the cardiac output. Lung capillaries have extremely high density (comprising of ~600 billion capillaries) (31, 32) with considerably low flow and narrow diameter (33). Lung capillaries originate directly from relatively large arterioles and not only from arterioles with precapillary sphincters (34), therefore, they are less protected from increases in pulmonary arterial pressure and prone to edema formation (35). In contrast to the systemic circulation, pulmonary circulation responds to hypoxia by vasocontriction and not with vasodilation (36). Pulmonary microvessels temporarily entrap polymorphonuclear leukocytes (PMNs) during a physiological process called PMNs margination, whereby 2-3 times higher number of PMNs reside in lung microvessels than in the systemic circulation, reaching dynamic exchange and an equilibrium between the two compartments (37). Unlike systemic circulation, PMNs emigration into the alveolar space takes place not only in postcapillary venules, but also through the capillary network (38) and larger arteries (39). Another unique feature of the lung microvasculature is constitutive expression of adhesion molecules (P-selectin and ICAM-1) (40–42). Nonetheless, typical intravascular rolling of PMNs was not reported in lung capillaries (43), and the role of classical adhesion receptors involved in neutrophil recruitment (selectins and integrins) remains to be debate in the inflamed lungs. As opposed to the systemic circulation, PMNs adhesion occurs through both CD11/CD18-dependent and -independent ways, depending on the cause (e.g. the source of infection) (44, 45). In addition, the role of dipeptidase-1-dependent PMNs adhesion has been recently reported (46). Therefore, it is important to optimally model pulmonary vasculature in the experimental set ups and choose an appropriate method to examine changes induced upon acute injuries. In further chapters of this review, we provide an overview about possible *in vitro* models addressing this complex issue.

**Figure 1:**
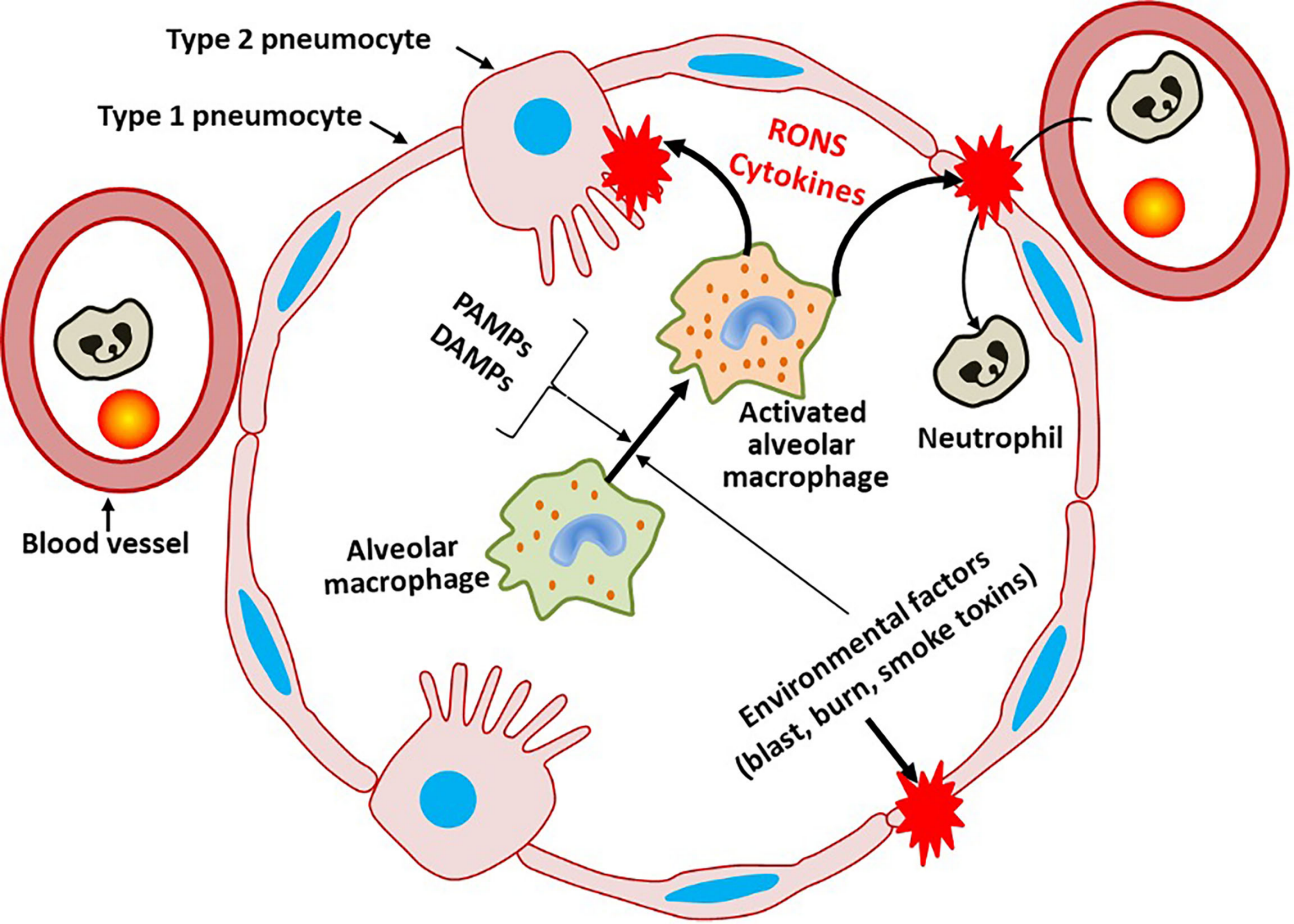
General mechanisms damaging pulmonary barrier. Infection *via* pathogen-associated molecular pattern (PAMPs) or damage-associated molecular pattern (DAMPs), the latter released due to physical damage of pneumocytes, activates alveolar macrophages (AMs) and later other immune cells migrating into alveoli. Excessive activation of AMs leads to the development of lung injury due to excessive generation of reactive oxygen and/or nitrogen species (RONS), cytokine storm and pro-coagulant activity.

**Figure 1** provides an overview on general mechanisms of pulmonary barrier damage.

## *In vivo* examination of the pulmonary microcirculation

Direct assessment of pulmonary microcirculation in septic patients is difficult to implement, thus suitable *in vivo* models are used to analyze several aspects of sepsis-related pulmonary dysfunction. *In vivo* examination of changes in microcirculation of the whole lung can be conducted by several methods including micro-computer tomography (CT) (47), and dynamic approaches such as single photon emission computed tomography (SPECT) (48) or microsphere techniques (49). If acquisition of spatiotemporal changes in circulatory and cellular inflammatory features of the microcirculatory compartment are of interest, different forms of intravital microscopy (IVM) could probably be a tool of choice (50, 51). These methods are based on conventional, one/two-photon or confocal fluorescence microscopic imaging methods as well as polarized light- and sidestream dark-field-based approaches in nearly all experimental animal species. During examination of the lung with IVM, observation of superficial microcirculation network of subpleural alveoli can be made possible *via* a surgically implanted thoracic window approach (52) supplemented with suctioning devices for stabilization purposes (53, 54). Yet, pulmonary motions and the beating heart cause fluctuations in vascular pressure and motion artifacts. Pulmonary movements could be overcome by taking recordings during end-expiration or after cessation of mechanical ventilation (55). Although the penetration depth of IVM methods is limited (<100 μm) (54), time-wise changes in microvascular diameters and capillary recruitment (55, 56), in cell-to-cell interactions (57–59), as well as alterations in endothelial glycocalyx (GX) integrity (60, 61) can be detected. Once IVM is combined with other methods, e.g. optical coherence tomography or multispectral oximetry, alveolar/airway dynamics (62, 63) and alveolar dynamics in parallel with capillary perfusion (55) can also be examined. Furthermore, subcellular process can be observed with two-photon microscopy at high resolution (54).

Leukocytes represent a particularly important first line of defense against airborne pathogens in the lungs. To date, IVM provides one of the best real-time methods to assess PMNs-endothelial and PMNs-platelet-endothelial interactions with regards to physiological PMNs margination (57, 64, 65). IVM-based studies have revealed the impact of leukocyte-platelet interactions (66) and their role in endothelium activation in the lungs (67, 68).

Although current experimental models are incomplete to recapitulate the major features of an acute lung injury and its clinical manifestation, the ARDS (69), the microcirculatory manifestations, ventilation- and sepsis-induced acute lung injury have been thoroughly examined in several studies. Numerous reports highlighted the impact of ventilation on microcirculatory processes in the lungs under experimental circumstances. Accordingly, respiration with high positive end-expiratory pressure (PEEP) values cause deterioration in capillary perfusion (56), and high respiration volumes evoke an exacerbation of endothelial-inflammatory cell interactions (70, 71). Ventilation-induced lung injury is also associated with the formation of neutrophil extracellular trap (NET) or NET-osis (72). Furthermore, increases in the lung microvascular pressure have been shown to increase cytosolic and intramitochondrial Ca^2+^ levels of endothelial cells (64, 73) with subsequent increase in pro-inflammatory cytokines and endothelium-derived adhesion molecule expression (64, 74). Looney et al. recently suggested that alveolar epithelium and vascular endothelium form a cellular syncytia favoring the spread of Ca^2+^ signal in a vectorial manner so as to achieve inflammatory communication in the multicellular environment within the lung, in response to mechanical forces (50).

Sepsis models are frequently used as acute lung injury models, and their relevance is marked by the fact that ARDS is a critical prognostic factor for mortality of clinical sepsis (75). In sepsis, lower pulmonary infections represent the leading cause of death (76). Further, diminished functionality of the microcirculatory perfusion is associated with increased mortality in septic patients. Notably, the ProCESS trial demonstrated an association between vascular density and De Backer score of sublingual microcirculation at 72 hours with 60-day mortality in septic patients (77). In experimental sepsis, CD11b/CD18-dependent PMNs accumulation in lung microvessels with a resultant mismatch in pulmonary ventilation-perfusion ratio could be visualized using IVM (59). Moreover, a link between heparanase-dependent process and the increase in PMNs adhesion has been demonstrated with IVM in the lungs of septic patients.

Moreover, IVM method allows the examination of endothelial surface layer GX which is in direct contact with the blood (78). Schmidt et al. showed that GX in the pulmonary endothelial cells appears to be thicker than in the systemic circulation, and is more vulnerable to sepsis than in other organs (60). Indeed, experimental endotoxemia/polymicrobial sepsis was associated with the degradation of GX (61). The relevance of these findings is underlined by observations whereby shedding of GX constituents from the injured endothelia into the blood stream was found to be important biomarker of pulmonary failure in patients undergoing septic shock (79). Elevated plasma levels of GX degradation products were also demonstrated in SARS/CoV/2 patients at a severe stage of the disease, and these changes were accompanied by GX damage in sublingual microvessels (80).

In summary, the functionality of the pulmonary microcirculation can be examined by several sophisticated methods of which IVM seems to be the method of choice for examination of pulmonary microcirculatory barrier upon lung injury. Despite an increasing understanding on the importance of microcirculatory changes in the progression of pulmonary diseases, so far, no specific microcirculation-targeted therapy reached a clinical significance. Nonetheless, real-time monitoring of disease progression and pulmonary microcirculatory manifestations upon therapeutic interventions may represent an important tool towards successful treatment of acute lung injury.

## Measurements of pulmonary mechanics in animal models

The structural extensity of airway epithelial cells and their crucial functions in the pulmonary barrier highlight the need to perform a comprehensive assessment of pulmonary mechanics in the *in vivo* studies. Animal models cover a wide range of pathologies including pulmonary fibrosis (81), smoke-induced chronic obstructive lung disease (82), neonatal chronic lung disease (83, 84), ventilator-induced lung injury (85, 86), viral and bacterial infections (86, 87). These models involve both airway and parenchymal alterations forming a complex scenario. Although the airway and lung parenchymal compartments are structurally connected, and operate together to maintain the pulmonary mechanics (88), characterizations of airway/parenchymal mechanics in terms of resistance (R) and elastance (E, the reciprocal of compliance) remained underrated.

Methodological precision and non-invasiveness tend to oppose each other, and this is referred to as the “phenotyping uncertainty principle” (89). At one extreme, the popular whole-body plethysmography in unrestrained animals was suggested to measure airway reactivity (90); however, this method has been shown to reflect more the changes in breathing pattern than the mechanical properties of the lungs (91). In contrast, the low-frequency oscillometry technique (LFOT) (92), which is considered the most sophisticated and selective assessment of the airway and tissue mechanics to date (93, 94) requires anesthesia and tracheostomy or intubation/cannulation of the trachea.

There are several other methods overviewed (89, 94). A robust traditional approach in mice (95) is the fitting of the transpulmonary pressure to a multiple-linear model of flow- and volume-dependence to obtain values of R and E. The main limitation of this technique is that the airway and tissue resistances are combined in the value of R. Attempts have been made to implement the widespread human pulmonary function tests such as the measurement of forced vital capacity (FVC) and forced expiratory volume in 1 s (FEV1) in small animals (96). However, the value of this implementation is dubious as increasing evidence challenges the sensitivity and structural specificity of the FEV1/FVC test (97).

LFOT employs small external pressure or volume oscillations during apnoeic intervals to estimate respiratory impedance (Z) at multiple frequencies covering the respiratory rate of the species (92, 98). The “constant-phase” model (CPM) of lung mechanics (92) is fitted to the measured Z values to obtain the parameters of airway resistance (Raw) and inertance, tissue damping (G) and elastance (H). The unique feature of this model is that G and H characterize the viscous losses and energy storing, respectively, of the respiratory tissues over a wide range of breathing and oscillation frequencies. The CPM has been validated in multiple studies using different resident gases, demonstrating the clear separation of the airway and tissue compartments. It has also been shown that non-uniform behavior of the lungs lends a virtual component to G (99). Extension of the CPM to express inhomogeneity *via* distributions of peripheral airway resistances and/or parenchymal units (100–103) has added further flexibility to cover diverse pathological alterations in lung mechanics. A large number of studies have contributed to establishing normative values for the CPM parameters in different species (93). Over 4 decades of body weight from 2-wk-old mice to adult humans, Raw, G and H arrange on the same trajectories and the log-log plots display high correlation coefficients (r^2^ = 0.91-0.98), while the G/H ratio expressing the mechanical efficiency or hysteresivity (103) of the lung tissue remains at the level of ~0.2, largely independently of the lung size (93). Thus, the LFOT-CPM method can be suggested as a universally accepted approach, yet with a parsimonious number of parameters, to evaluate lung mechanics in animal models of pulmonary diseases.

Majority of the methods on lung mechanics are used on the whole respiratory system. Therefore, the results include the mechanical impedance of the chest wall, unless thoracotomy or oesophageal pressure measurement is performed. In larger mammals, chest wall properties may mask the changes occurring in the lungs, whereas in small rodents with compliant chest wall structures, especially in mice, this error may be negligible.

Finally, in many models of pulmonary disease the temporal dynamics of the development of pathological changes may become a primary aspect. Repeated studies are technically feasible in mice (104) and rats (105) and they can be combined with high-sensitivity lung function techniques to properly address the questions raised in the animal models.

## Alveolocapillary *in vitro* model (air-liquid interface)

In the past years, several *in vitro* models of the air/blood barrier were developed. The principal aim was to enable the use of a solid method for investigations of the air/blood barrier and determine the potential impacts of harmful exposures. In contrast to submerged 2D culture models, the air/blood model mimics the air surface side of the alveolar epithelium. As cells of the pulmonary system show an environment-dependent differentiation behavior, it is crucial to mimic this air/interstitial environment. Airway cells cultured in the air-liquid interface (ALI) show significant morphology and barrier characteristics compared to the submerged culture condition (106). Therefore, ALI models are particularly exploited to investigate the impact of various exposures on the barrier integrity by using several immortalized cell lineages (107). In the ALI model, cells are grown on transwells or biological scaffolds (108), where the basolateral side is in contact with cell culture media, whereas the apical side of the cells is exposed to the air (109). The currently used cell lines are either of alveolar origin (*A549* (110), *hAELVi* (111), *hAEpC* (112), and *NCI-H441* (113), H358 (108), HCC827 (114) or of bronchial origin [*Calu-3* (115), *BEAS 2B* and *16HBE 14o*^-^ (116), primary human airway epithelial cells (pHAECs) (117)]. Interestingly, pHAECs were successfully grown in miniaturized transwell plates (118). These primary human cells are superior to immortalized cells in terms of cell behaviour. However, they lack comparability due to donor-dependent attributes. This limitation was overcome by ALI differentiation of human-induced pluripotent stem cells into AEC II (119). Given the lack of blood supply in ALI model, modification made by co-culturing capillary endothelial cells, including HPMEC (114, 120) and EA.hy 926 (121) with AECs to optimally recapitulate the air-blood barrier (122). Combination of both endothelial and alveolar components allows optimal assessment of the cell-cell interaction during pulmonary inflammation (114, 123). For instance, the impact of bacterial component lipopolysaccharide (LPS) (124, 125) or human inflammatory cytokines like initerferon (IFN) or TNF (118) on the barrier is evaluated by ALI model. In such models, it is possible to include leukocytes in addition to the alveolar cells (126, 127) and endothelial cells in a triple culture to optimally mimic disruptive barrier conditions during inflammation (120, 128).

Although 2D monolayers or well-differentiated airway epithelial cells in ALI are commonly used to investigate pulmonary inflammation (129, 130), these models undermine the impacts of inflammatory cells recruiting to the site of inflammation. To close this gap, primary human vascular endothelial cells (HMVEC-LB1), human white blood cells (WBC), and their co-cultures can be used to evaluate their inflammatory responses to various inflammatory stimuli (131). HMVEC-LB1 cells can be applied as an *in vitro* model to examine pro-inflammatory responses to inflammatory trigger by measuring the production and/or release of IL-6, soluble intercellular adhesion marker (sICAM)-1, or soluble vascular cell adhesion marker (sVCAM)-1 (131). Furthermore, an alveolo-capillary barrier *in vitro* model consisting of a co-culture system of human distal lung epithelial cells and HMVECs was established (113, 132) to assess epithelial-endothelial interactions in context of acute lung injury (113). Functional cellular junctions are formed in these models by a tight epithelial barrier similar to *in vivo* conditions. More specifically, the *in vitro* co-culture system consist of monolayers of human lung epithelial cell lines (A549 or NCI H441) and HMVECs on opposite sides of a permeable membrane. Although A549 failed to show sufficient differentiation with respect to formation of a tight epithelial barrier with intact cell-cell junctions (113), the co-cultures of NCI H441 and HPMEC established differentiated monolayers after stimulation with dexamethasone by forming tight junctional protein, ZO-1 and the adherens junction protein, E-cadherin (113). Furthermore, the model induced a polarized epithelial cell monolayer with typical junctional structures as confirmed by transmission electron microscopy. Taken together, such a co-culture system constitutes a suitable *in vitro* model to examine e.g. functional barrier epithelial, and epithelial/endothelial interactions in the pathogenesis of acute or toxic lung injury and inflammatory lung diseases.

## *In vitro* models of pulmonary inflammation and barrier function

Simple monoculture studies have enabled researchers to understand the abnormal phenotypes of lung cells in diverse pathologies. For example, 2D models are well established for the research of lung function, as they can fully differentiate to airway cells as well as AEC I-like and AEC II-like cells (133). Although 2D cultures are well established in pulmonary epithelial barrier studies, *in vitro* co-culture models are advantageous in recapitulating the complex structure and function of native lung by mimicking the multicellular interactions. Such co-culture models aiming to assess cellular communication in the lung include conditioned medium-exposure experiments, epithelial-mesenchymal co-cultures, and several 3D co-culture models. 3D cultures can be further differentiated into so-called “human lung organoids” (HLOs). These lung progenitors can give rise to proximal airway-like structures, as well as to alveolar cell types (134, 135). Furthermore, biological and synthetic scaffolds like decellularized rat and human lung slices or whole organs can be used to develop a more functional lung cell response to inflammation (136–138). These models provide insights into cellular communication either directly *via* assessment of cell-to-cell interactions or indirectly *via* assessment of the release of soluble mediators which mimic *in vivo* conditions in the pulmonary system. Nevertheless, various models require careful assessment of cell entities as well as used culture media to ascertain the viability and functionality of co-cultured cells.

### 2D *In Vitro* Models

It is already known from simple co-culture experiments that the cellular constituents of the alveolar-capillary wall may be key determinants in the recruitment of PMNs to the lung through the generation of chemotactic agents including IL-8 (139). This IL-8-mediated recruitment of PMNs occurs *via* AM-derived chemokines, TNF, and IL-1 that induce IL-8 release from AEC II cells (139). 2D models have demonstrated potential cell-to-cell communication circuits that are important between AMs and AECs during the recruitment phase of acute lung inflammation. Furthermore, it was reported that unique location of the pulmonary fibroblasts (PFs) allows communication between the vascular compartment and alveolar airspace in a bidirectional fashion, which is essential for recruitment of inflammatory leukocytes into the lung (140). It is reported that PF-derived IL-8 expression in co-culture was first dependent upon activation of the AMs by LPS and subsequent release of macrophage inflammatory mediators (140). Thus, interaction between AMs and PFs may be important in the pulmonary barrier, resulting in the generation of IL-8 and subsequent recruitment of inflammatory leukocytes (140).

Since the AECs can release several chemokines fundamental to both inflammatory and immune responses, a simple monolayer culture using A549 cells which are representative of alveolar epithelium can be used to mimic inflammatory conditions (141, 142). A549 cells can be exposed to cell culture medium that has been conditioned by tobacco smoke, known as submerged culture, to study different lung pathologies (143). This model can be used to mimic lung pathologies in COPD, as a tobacco smoke-related pulmonary disease, which is characterized by heterogenous pathologies including chronic inflammation involving different cells, mainly epithelial cells, macrophages, neutrophils, and CD8 lymphocytes (143, 144). Exposing A549 cells to tobacco smoke-conditioned medium provoked the release of chemokines by lung epithelial cells (145). Such simple *in vitro* epithelial cell culture model may allow the initial evaluation of novel anti-inflammatory compounds for the treatment of e.g. COPD. Furthermore, this approach may facilitate replacement of animal experimentation in novel drug screening for COPD (145).

Other *in vitro* models to study e.g. the endothelial-dependent mechanisms that mediate leukocyte recruitment and address leukocyte adhesion or in depth biochemical analyses during an inflammatory response are based on the isolation of murine vascular endothelial cells from the lung and heart and subsequent *ex vivo in vitro* culture (146). The isolated endothelial cells with this method provided high purity rates (85-99%) and retained their functional differences, including constitutive and cytokine inducible adhesion molecule expression and chemokine production (146).

### Co-Culture Models in ALI

The co-culture models with two cell types have been extended for further pathology-relevant cell types. AMs demonstrate a specific functionality, as they inhabit a unique microenvironment with high oxygen levels and exposure to external hazards. Thus, those cells have been integrated in a multicellular co-culture systems of the respiratory tract to unravel mechanisms underlying pulmonary inflammation (122). Kasper et al. have studied the immunological impact of distinct macrophage phenotypes that were seeded on to the epithelial layer of an established *in vitro* air-blood barrier co-culture, consisting of alveolar epithelial cells and microvascular endothelial cells on the opposite side of a transwell filter-membrane (122). Of note, healthy AMs display an anti-inflammatory phenotype, that prevents hypersensitivity and maintains tissue homeostasis in the alveoli (147). The physiological functions of AMs are provided by three differentially polarized AMs types, including classically activated M1 and alternatively activated wound-healing M2 and regulatory M2 macrophages (147, 148). LPS challenge can be applied to evaluate activation of AMs by measuring the release of pro-inflammatory molecules e.g. IL-8 and sICAM upon stimulations (147, 149). M1 macrophages were used in these models to provoke severe inflammatory-like response of the air-blood barrier co-culture, whereas “non-inflammatory” M2 macrophages may be used to establish a quiescent, physiological *in vitro* air-blood model (122). These complex co-culture models of alveolar epithelial-endothelial cells and macrophages provide a responsive *in vitro* model of the air-blood-barrier that mimics inflammatory features similar to *in vivo* condition (122). Nevertheless, it is still challenging to identify the most appropriate cell line as well as ALI culture method to be used in these models. Recently, studies with the CuFi-1 and the NuLi-1 (healthy bronchial epithelial cell lines) cells have demonstrated that both cell lines fully differentiate in ALI culture with significant mucus production and secretion, expose an inflammatory response characterized by IL-6 and IL-8 production, and functional tight junctions (150). Thus, *in vitro* ALI models with these cell lines may be promising to investigate the inflammatory condition in air-blood barrier co-culture models.

### 3D *In Vitro* Models in ALI

3D cultures are superior to 2D models as they optimally mirror the complex functional structure of a native lung. In 2008, the 3D *in vitro* model of the human airway was generated by using a co-culture of normal human bronchial epithelial cells (HBEs) and normal human fibroblasts for the health risk assessment of carbon nanotubes on the human respiratory system (151). In this model fibroblast-embedded collagen I gels were used in combination with bronchial epithelial cells in ALI. 3D model was generated by adding a mixture of collagen and normal human lung fibroblasts (NHLFs) to the underside of a transwell polyester membrane, whereas HBE cells were seeded on top of the polyester membrane (151). Furthermore, complex tetra-culture systems combining lung alveolar epithelial cells, endothelial cells, macrophages, and mast cells in 3D have been developed to accurately model inflammatory condition *in vitro* (152).

Another *in vitro* ALI 3D human airway model includes airway cells from patients undergoing surgical polypectomy and MucilAir (Epithelix Sarl, Geneva, Switzerland) airway epithelia cultured in transwell inserts (153). Here, a range of different parameters such as cytotoxicity, cell barrier integrity, viability, morphology, ciliary beating frequency, mucociliary clearance, and cytokine release can be analyzed for predictive accuracy for e.g. respiratory toxicity. In addition to ALI 3D human airway models, various 3D airway tissue models based on a collagenous scaffold were developed (154). The advantage of this system is that the scaffold contains a basement membrane (155) that maintains natural barrier function. Complex co-cultures of primary epithelial cells (156) primary fibroblasts, and endothelial cells (114) can be built up on this scaffold. These complex models have been successfully combined with the immune cells to investigate of infection biology (108).

Another interesting approach to generate 3D lung structures is an ultrasound trap-based technique to rapidly form cell aggregates known as spheroids (157, 158). Here, different cell types including A549 can be applied to study e.g. different receptor expression and cytokine response (157). 3D spheroid cultures “mimic” the typical *in vivo* cell responses to e.g. endotoxin during the development of inflammation and may be a superior *in vitro* model compared to other *in vitro* models to study inflammatory conditions (157).

### 3D Bioprinting *In Vitro* Models

The 3D bioprinting provides a promising technique to generate viable and functional tissues to study organ functions under physiological and pathophysiological conditions (159). In this model cells are printed or more specifically dispensed by automated systems on specific substrates or materials, whereby mixtures of the cells and extracellular matrix (ECM) components are referred to ‘bioinks’ (160). The composition of biosubstances has significant impact on the biofunctionality and printability of bioinks to generate 3D structures (161). The technology of bioprinting is divided into the methods of micro-extrusion, droplet-based printing, and laser-assisted printing (161).

In the first attempt, Horváth et al. have established a double layer system comprising endothelial cells, epithelial cells and Matrigel^®^ by 3D bioprinting to generate the air-blood tissue barrier with a proven viability of cells (162). More recently, Park et al. developed an 3D model using tracheal mucosa-derived decellularized ECM (tmdECM) and human dermal microvascular endothelial cells (hDMECs) to induce blood vessel formation, lung fibroblasts (LFs), and human tracheal epithelial cells (hTEpCs) (163). The vascular network in this setup responded to inflammatory stimuli by enhanced expression of adhesion molecule ICAM-1 that maintained for several weeks (163). This approach seems to be a promising 3D bioprinting technique to analyze the pulmonary barrier under acute inflammatory conditions by an application of pro-inflammatory stimuli.

### Stem Cell-Based Lung Epithelial Cells Models

Stem cell-based *in vitro* lung models gained more attention in the past years, especially in the era of SARS-CoV-2 pandemic. In fact, stem cells seem to display one major therapeutic approach to beat SARS-CoV-2 infections, as their effectiveness is currently examined in multiple clinical trials (164). Besides their therapeutical potential, stem cell-based models also offer the valuable possibility to gain detailed insights into mechanisms underlying cellular dysfunction leading to alveolar barrier damages.

Using several progenitor lung epithelial cells, several models have been established like 2D and partially transferred to ALI models, 3D models, and recently multigerm 3D models (165) as well as *human pluripotent stem cell-derived lung organoids* (165, 166). So far, 2D models seem to be the most widely used pulmonary stem cell models (167). 2D pulmonary stem cell models are used for functional analyses, as they can generate a monolayer of proximal airway cell types as well as differentiated AEC I-like and AEC II-like cells (133, 168). Like the immortalized cell lines used in pulmonary research, the monolayers formed by bronchial-like or alveolar-like-cells were transferred into ALI conditions (119, 133, 169) offering the possibility to combine the advantages of both techniques. As a relevant drawback of the 2D models, they take a relatively long culture period at the ALI, and thus, are highly time consuming. Initially, they offer a mixture of different cell types, which are specified into selective lung progenitors (170). Nevertheless, stem cell-based models represent a huge step towards the transferability of findings into clinical settings. By combining progenitor stem cells and 3D models, the interplay of several differentiated alveolar cells can be evaluated in a human-comparable chemically and physically microenvironment (136, 171).

### Human Lung Organoid Models

3D stem cell-based models were further improved to lung organoids, generating even higher clinical applicability (172, 173). Organoids are nowadays widely defined as 3D structures grown from stem cells and displaying organ-specific cell types as well as self-organizing and self-renewal capacity (174). These highly complex “mini-organs” can be built from various stem cells including human pluripotent stem cells (hPSCs). Therefore, the assessment of a patient-specific inflammatory influences and pathogen-sensitivity or reliance is now a reachable goal (175). HPSCs are developed into lung lineages in a process called direct differentiation, which mimics natural signals to induce different steps of differentiation: gastrulation, patterning, and specification (176). In addition to hPSCs-derived human lung organoids (HLOs), human airway basal cells serve as progenitor cells to form lung organoids (177, 178), increasing the cell sources for the generation of lung organoids.

Lung organoid models provide the opportunity to assess infection and mechanical stress at different developmental stages (179). HLOs can be used to evaluate the mechanical lung compression *in utero* during the pseudo glandular stage (180). Furthermore, lung organoids support the growth of viruses in the absence of selective pressure and by adapting to culture conditions, thus, can be used in pulmonary viral infection studies. For example, lung infection at different degrees of prematurity can be assessed by HLO model e.g. to explore impacts of early viral infection on lung development. 3D alveolar organoids have also been used to assess pathomechanisms of emerging viral infections like SARS-CoV-2. Most recently, an alveolosphere culture system of human AEC II was established from alveolar-progenitor cells (181). These cells maintained their cardinal features *in vitro* like self-renewal and differentiation into AEC I as well as surfactant production (181). As the organoid AEC II cells highly express SARS-CoV-2 entrance receptor angiotensin-converting enzyme receptor type 2 (ACE-2), similar to *in vivo* condition, they were used in SARS-CoV-2 studies (178, 181). Another study examined a mass spectrometry analysis of infected AEC II cells, derived by a hPSC based alveolosphere model (182). Tight junction proteins like claudin-18a were significantly decreased in infected AEC II cells, displaying an impaired apical barrier which might be causative for the lung edema formation as observed in ARDS (182). Besides, alveolosphere organoids served already to examine the negative effects of external factors on the alveolar epithelium. Of note, alveolar organoids exposed to ambient particulate matter showed enhanced expression of ACE-2, increasing the susceptibility to SARS-CoV-2 (183). Taken together, HLO can serve as a crucial *in vitro* model to understand cellular mechanisms as a response to toxic stimuli as well as to pulmonary infections (181). Nevertheless, HLO model needs special handling, cultivation improvements, and further research.

### Lung-on-a-chip (LOAC) *In Vitro* Models

Generation of tissue models showing various physiological functions and minimal numbers of cells is necessary. The use of minimal number of cells is advantageous due to the lack of cell donors and functional cell lines and the variable composition of necessary hydrogels for efficient cell culture of organoids, stem cells or embryonic cell cultures (184, 185). Furthermore, it is well known that physiological stimuli, such as stretching (186) fluid shear stress (187) or air contact (188), significantly alter specific functions, especially in airway cells (188). For this, few labs started to develop microfluidic systems for the culture of one or more tissue models (189). One of the first and most successful developed models is the “lung-on-a-chip” (LOAC) (190) that Ingber’s group reported in 2010. This model was based on a biomimetic microsystem that reconstitutes the critical function of alveolar-capillary interface of the human lung (190) by co-culturing alveolar and endothelial cells on a flexible membrane. The breathing function is mimicked by applying a regular vacuum in the device. Furthermore, this vacuum moves the membrane and induces stretching of the cellular layer, enabling long term functional culture of the lung model. Such innovative bioengineered LOAC *in vitro* models hold future promises for understanding abnormal pulmonary homeostasis and assessing therapeutic strategies in the pulmonary diseases (191, 192). Of note, LOAC models are used do simulate early *Mycobacterium tuberculosis* infection of the lung and reproduces complex integrated organ-level responses to bacteria and inflammatory cytokines released into the alveolar space (193). LOAC models are also successfully used to investigate mechanisms underlying pathogenesis of acute respiratory SARS-CoV-2 infection. In this LOAC model, a co-culture of human alveolar epithelium, microvascular endothelium and circulating immune cells was established under fluidic flow. Transcriptional analyses revealed an activated innate immune response in epithelium and cytokine-dependent pathways in the endothelium upon infection with SARS-CoV-2 (194).

The final aim for using these models is development of low-cost alternatives to animal and clinical studies for drug screening and toxicology applications. Nevertheless, the combination with other organs is necessary and under development to address the multiple organ interactions (195).

### Precision-cut Lung Slices (PCLS) Models

The high-throughput precision-cut lung slices (PCLS) model includes culturing fine sliced lung tissue explants collected from patients with pulmonary diseases or experimental animal models by either submerging these slices in culture medium or in ALI (196). The PCLS model was initially developed to largely screen the immunotoxicology impacts of various therapeutic compounds as well as environmental exposures on the lungs (197). In obvious contrast with other *in vitro* models, in PCLS the lungs preserve their structural and cellular compositions which is advantageous to investigate cell-cell interaction in the lung microenvironment (197). Furthermore, the extracellular matrix (ECM) which acts as a scaffold reinforcing tissue architecture remains intact in the PCLS (196), making it a suitable model to investigate lung repair and regeneration upon injury. Indeed, a recent study reported that PCLS models are ideal to monitor alveolar regeneration, which is impaired in acute lung injuries, particularly in ARDS (198). In line, PCLS has been successfully used in a study to investigate alveolar-specific regeneration after injury by acid (199). However, the blood perfusion is absent in PCLS models, which causes the exclusion of recruited immune cells from the circulation to the lung (200). A solution for this limitation is to co-culture PCLS with peripheral blood mononuclear cells (201). Moreover, due to the incisions for cutting the lung tissues to fine slices, it is not expected to be an ideal model for investigating lung barriers as barriers may be locally damaged. Another disadvantage of PCLS is overlooking the interaction of other organs with the lung. For instance, gut-lung interaction is well-known to impact lung microbial compositions (202) as a factor that may affect the barriers (203) which is missed in PCLS models. Although short-term viability of cells in culture is another issue with this model, recent studies found that cryopreservation or coating with hydrogels may increase the culture window from a few days to several weeks (204, 205). In summary, PCLS models have shown limited applications for investigation of lung barrier integrity during acute lung injury and may only be used to monitor cellular and ECM regeneration upon lung injury.

**Table 1** summarizes *in vivo* and *in vitro* models of pulmonary barrier to study acute inflammatory diseases, and **Table 2** provides the benefits and limitations of different *in vitro* models.

**Table 1:** *In vivo* and *in vitro* models of pulmonary barrier to study acute inflammatory diseases.

*In vivo* models	*In vitro* models
Analysis of pulmonary microcirculation micro-computer tomography (CT) (47) single photon emission computed tomography (SPECT) (48) microsphere techniques (49) intravital microscopy (IVM) (50, 51) combinable with e.g. optical coherence tomography (OCT) (62, 63), multispectral oximetry (55), or two-photon microscopy (54) Analysis of pulmonary mechanics whole-body plethysmography (90) low-frequency oscillometry technique (LFOT) (92) fitting of transpulmonary pressure to a multiple-linear model of flow- and volume-dependence in mice (95) measurement of forced vital capacity (FVC) and forced expiratory volume 1 (FEV1) (96)	Analysis of pulmonary inflammation and barrier function air-liquid interface (ALI) models (106–110) 2D *in vitro* models, e.g. using AMs, AECs and pulmonary fibroblasts (PF) (139, 140) co-culture models in ALI, e.g. using AMs (122) 3D *in vitro* models in ALI ALI 3D human airway models (151–154) 3D spheroid cultures by ultrasound trap-based technique (157, 158) 3D bioprinting *in vitro* models (162, 163) 2D pulmonary stem cell models (167) human lung organoid models (172, 173, 181) lung-on-a-chip (LOAC) model (190) precision-cut lung slices (PCLS) models (196–199)

**Table 2:** Benefits and drawbacks of different *in vitro* lung models.

Model	Benefits	Drawbacks
Air-liquid interface (ALI)	possible with primary cells, immortal cell lines and hPSCs mimics the air surface side of the alveolar epithelium available as 2D, 3D models as well as co-culture	lack of standards by primary cell lines different metabolic conditions by immortalized cell lines
Human lung organoids (HLOs)	different cell sources possible more clinical applicability	needs special handling and cultivation improvements
Lung on a chip (LOAC)	minimal numbers of cells necessary allows stretching of the cellular layer	high costs, limited availability
Precision cut lung slices (PCLS)	preserve structural and cellular composition of the lung extracellular matrix remains intact	lack of blood perfusion short term viability

## Conclusions

Extensive understanding of mechanisms underlying pulmonary barrier dysfunction is pivotal for the development of therapeutical strategies in acute lung injuries. To better understand molecular mechanisms leading to both deterioration and consecutive dysfunction of pulmonary barrier several *in vitro* and *in vivo* models have been developed. In this review, we highlighted the development and applications of these models, including 2D mono- and multiple cell-types, co-culture models, 3D air-liquid interface models, 3D bioprinting models, lung-on-a-chip, precision-cut lung slices, and lung organoids. These models may improve our understanding of the complex pulmonary homeostasis under regular and pathophysiological conditions by mimicking the *in vivo* environment. Given the increasing number of studies applying these models, they represent valuable platforms to assess cell-to-matrix, cell-to-cell interactions as well as the role of exogenous and endogenous inflammatory or danger associated molecules in lung damage and repair mechanisms. The use of such complex 3D models will further improve the incorporation and assessment of specific cellular phenotypes and humoral factors by sustaining cell functionalities in a manner that resembles the *in vivo* conditions to elaborate their impact on morphology, toxicity, viability, barrier integrity, ciliary beating frequency, mucociliary clearance, as well as cytokine release. The understanding of complex interactions between lung structural cells, (immune) environment, and cellular matrix in the lung micromilieu will provide further options to investigate novel therapeutics for targeting different pathogenic mechanisms in acute lung injury.

## Data Availability Statement

The original contributions presented in the study are included in the article/supplementary material. Further inquiries can be directed to the corresponding author.

## Author Contributions

All authors listed have made a substantial, direct, and intellectual contribution to the work, and approved it for publication.

## Funding

AVK was supported by the Ludwig Boltzmann Institute Traumatology. The Research Center in Cooperation with AUVA Austrian Cluster for Tissue Regeneration. ZH was supported by Hungarian NKFI-OTKA grant K128701. SG was supported by the Federal Ministry of Education and Research (BMBF), The STIMULATE Research Campus, grant number 13GW0473A. SMC was supported by the Federal Ministry of Education and Research (BMBF), grant number 03Z22JN12 to SC. BR was supported by the Federal Ministry of Education and Research (BMBF), The STIMULATE Research Campus, grant number 13GW0473A to BR.

## Conflict of Interest

The authors declare that the research was conducted in the absence of any commercial or financial relationships that could be construed as a potential conflict of interest.

## Publisher’s Note

All claims expressed in this article are solely those of the authors and do not necessarily represent those of their affiliated organizations, or those of the publisher, the editors and the reviewers. Any product that may be evaluated in this article, or claim that may be made by its manufacturer, is not guaranteed or endorsed by the publisher.
